# A150 MODERATE AGREEMENT IN ENDOSCOPIC DISEASE SCORING OF PEDIATRIC EOSINOPHILIC ESOPHAGITIS AMONG PEDIATRIC GASTROENTEROLOGISTS IN CANADA

**DOI:** 10.1093/jcag/gwac036.150

**Published:** 2023-03-07

**Authors:** A Chung, D Ashok, V Avinashi, J Barkey, K Bortolin, D Burnett, B Chen, J Critch, É Drouin, J Griffin, J Hulst, M Marcon, A Martinez, R Persad, M Sherlock, H Huynh

**Affiliations:** 1 University of Alberta, Edmonton; 2 University of Western Ontario, London; 3 Canadian Pediatric EoE Network, -; 4 BC Children's Hospital, Vancouver; 5 University of Ottawa, Ottawa; 6 SickKids, Toronto; 7 Dalhousie University, Halifax; 8 University of Saskatchewan, Saskatoon; 9 Memorial University, St. John's; 10 Université de Montréal, Montreal; 11 University of Manitoba, Winnipeg, Canada; 12 McMaster University, Hamilton, -

## Abstract

**Background:**

Endoscopy is an important tool in assessing the severity of gastrointestinal diseases including Eosinophilic Esophagitis (EoE). Agreement regarding endoscopy outcomes is important when using tools such as the Endoscopic Reference Score for EoE (EREFS).

**Purpose:**

Our goal was to determine interrater and intrarater agreement of EREFS among Canadian pediatric gastroenterologists.

**Method:**

Survey-based study of interrater and intrarater reliability amongst pediatric gastroenterologists with interest in pediatric EoE. Participants were sourced from the Canadian Pediatric EoE Network. Participants were asked how many years of training they’ve had with endoscopy for pediatric EoE and their comfort in disease scoring for pediatric EoE. Pediatric EoE cases were identified from the pediatric EoE registry at the Stollery Children’s Hospital with an endoscopic video associated with each case. Participants were asked to score each video using the EREFS questionnaire for the proximal, middle and distal segments of the esophagus. 15 endoscopic videos were evaluated, with 3 cases provided each week over a period of 5 weeks. Additional data included ratings of the video quality and endoscopy quality. Of 15 cases, 12 were unique cases, distributed evenly in severity between no active disease to severe disease. 3 cases were repeated to assess intrarater reliability. The maximum grade of the proximal, middle and distal segments of the esophagus for each component endoscopic finding (edema, rings, exudates, furrows, strictures) were used for reliability calculations. Fleiss Kappa was calculated for all EREFS items and for each component endoscopic finding. Cohen’s Kappa was calculated to assess intrarater reliability.

**Result(s):**

Fifteen participants were recruited for the study. The participants had a median of 12 years (IQR: 7, 19) of clinical experience in endoscopy for pediatric EoE. The majority of participants were “comfortable” (i.e., 4 on 5-point scale) with EREFS scoring for pediatric EoE. Fleiss Kappa for all EREFS items was 0.481. For each component endoscopic finding (edema, rings, exudates, furrows, strictures), Fleiss Kappa was 0.365, 0.293, 0.548, 0.263, 0.445 respectively. Cohen’s Kappa had a median of 0.620 (IQR: 0.593, 0.704). The majority of raters rated video quality and endoscopy quality as “good” (i.e., 4 on 5-point scale).

**Conclusion(s):**

There is moderate interrater reliability in EREFS scoring for pediatric EoE. Interrater reliability was between fair to moderate for each component endoscopic finding. Intrarater reliability was good. This study shows there is room for improvement in disease scoring for pediatric EoE. This could be in the form of additional training, expert-defined conventions, or centralized reading which have reduced variability in endoscopic reporting for adult GI disease in past studies and could be used in a follow-up study to attempt to improve agreement. Additionally, incorporating EREFS into routine clinical practice may increase agreement amongst endoscopists.

**Please acknowledge all funding agencies by checking the applicable boxes below:**

None

**Disclosure of Interest:**

None Declared

